# Review of current knowledge regarding usage of pre-hospital heart rate variability and complexity in triage and added value for predicting the need for life-saving interventions

**DOI:** 10.1186/s12245-025-00967-4

**Published:** 2025-09-23

**Authors:** Christoffer B. Hedegaard, Kasper Iversen, Fredrik Folke, Morten Lock-Hansen, Carolina Malta Hansen, Jannik Pallisgaard

**Affiliations:** 1 Department of Clinical Medicine, Faculty of Health and Medical Sciences, University of Copenhagen, Copenhagen, Denmark; 2 Department of Emergency Medicine, Herlev and Gentofte, Gentofte, Denmark; 3 Department of Cardiology, Copenhagen University Hospital - Herlev and Gentofte, Gentofte, Denmark; 4 Copenhagen Emergency Medical Services, Copenhagen, Denmark; 5 Private Consultant Cardiologist, Copenhagen, Denmark; 6 Department of Cardiology, The Capital Heart Center, Rigshospitalet, Copenhagen, Denmark

## Abstract

**Background & aim:**

Analysis of heart rate variability metrics has shown added accuracy in pre-hospital trauma triage. These metrics include heart rate variability (HRV), meaning oscillations in the time interval between heartbeats, and heart rate complexity (HRC), which assesses intricate patterns of heart rate over time. This review article evaluates current knowledge regarding HRV and HRC and prediction of a subsequent life-saving intervention (LSI), an intervention executed by trained medical personnel to prolong the life of the patient. Our primary focus was on pre-hospital patients and the utility of HRV and HRC when added to existing trauma triage scores or vital signs such as heart rate (HR).

**Methode:**

A literature search was carried out by searching the MEDLINE database via the PubMed website for original research published in English from 2008 to 2023. The combinations of search terms applied yielded 18 original studies of which only six met our criteria. We included another study as it contributed original research beneficial to our article.

**Results:**

The studies showed a statistically significant increase in the ∆Area Under Curve (AUC) between 0.14 and 0.40 for predicting risk of LSI when adding the two heart rate variability metrics to existing trauma triage scores or vital signs such as HR. Calculation of HRV and/or HRC could be conducted using ECG recording hardware already accessible in most emergency pre-hospital settings with less ECG noise and therefore higher quality ECG data over time.

**Conclusion:**

Both HRV and HRC showed potential for increasing ∆AUC in predicting risk of LSI when added to existing risk triage scores. Calculation of HRV and HRC could potentially be conducted using a preexisting hardware in most emergency pre-hospital settings.

## Introduction/background

Trauma-related injuries constitute the leading cause of mortality in the United States for people under 35 years, accounting for approximately 36 million emergency department visits, 2.6 million hospitalizations, and more than 150,000 deaths in America annually [1]. Of those suffering from fatal traumatic injuries, up to 40% die before they reach the hospital [2]. Correct triage enables healthcare personnel to treat patients sooner, which has been proven to decrease mortality [3]. Thus, the ability to predict which patients could need an intervention which will directly prolong the life of the patient, preformed either pre-hospital or in the emergency department (a life-saving intervention (LSI)) [4], is therefore pivotal to ensure that the correct patients are transported to the hospital in a timely manner with minimal over- or undertriage [5]. An LSI, is therefore an endpoint that varies among the articles and lacks a universal definition, interventions included but are not limited to; cardiopulmonary resuscitation, needle decompression and endotracheal intubation.

Traditional triage is based on vital signs such as heart rate, blood pressure, respiratory rate, temperature, saturation, level of consciousness, and severity of injury. When vital signs are abnormal, it suggests an increased risk of mortality and consequently the use of LSI’s in the endeavor to prevent death [6]. Vital signs are the most commonly used variables in triage scores, even though multiple new studies state the limitations of these factors in trauma triage in predicting of LSI, such as patients suffering from blunt trauma or excessive bleeding [6, 7]. Thus, two new physiological factors are increasingly recognized for their importance in trauma triage; heart rate variability (HRV) and heart rate complexity (HRC) [4, 8].

The human heart is regulated by a complex network of signals from the autonomic nervous system, hormonal influences, and other physiological factors. These systems work together in a delicate balance to adjust heart rate according to the body's needs, such as physical activity, stress, or relaxation. HRV refers to the natural variation or so-called oscillations in the time interval between consecutive heartbeats. HRV is believed to be a marker of the autonomic nervous system's regulation of the heart. An increase in HRV is associated with rest, exercise, and good recovery, whereas a decrease in HRV is associated with stress and illness [9].

HRV and HRC are derived from ECG metrics, giving it the great advantage of reproducibility [10]. When analyzing ECG’s various analysis techniques can be utilized when calculating HRV. This includes; *time domain* (Standard Deviation of Normal to Normal R-R interval (SDNN)), *frequency domain* (Low Frequency (LF), High Frequency (HF) and Low Frequency High Frequency ratio (LF/HF)) and/or *entropy*(Sample Entropy (SampEn), Approximate Entropy (ApEn), Fractal Dimensions by curve Length (FD-L) and Detrended Fluctuation Analysis (DFA)) [11]. The most commonly applied analysis technique is SDNN, which an index of HRV [5]. Entropy analysis is applied when calculating HRC [12]. HRC refers to the intricate and nuanced patterns found within the variations of heart rate over time. Unlike a simple measurement of beats per minute, heart rate complexity examines the subtle fluctuations, waveforms and irregularities in the heart's rhythm, and reflects the dynamic interaction between autonomic systems in the body such as sympathetic and parasympathetic [13]. When HRC is calculated, a reduction in the entropy demonstrates a reduction in the complexity of these systems [14]. Multiple studies have shown that a reduction in HRC is associated with a higher mortality rate [13, 14].

Research suggests that both HRV and HRC can significantly enhance the accuracy of triage of prehospital patients, when added to existing scores, subsequently improving the chances of timely treatment and LSI’s [8]. These metrics could potentially provide insights into a patient's current physiological state, offering a more nuanced understanding than traditional vital signs. HRV and HRC analyses could be incorporated into trauma triage workflows and into already pre-existing vital signs monitors without the need to add new hardware [15]. This non-invasive triage method could potentially change triage in emergency medical response, leading to more effective allocation of resources and better patient outcomes [5].

This review, sought to assess the current knowledge regarding HRV and HRC in a pre-hospital setting and their ability to work in triage and prediction of risk of LSI.

## Methods

Original research articles published in English between 2008 and 2023 were identified through a literature search conducted on the PubMed website using the MEDLINE database. The studies retrieved are pertaining to HRV and/or HRC. The studies all analyzed ECG metrics to obtain HRV and/or HRC metrics. The studies investigate how HRV potentially can be indicators of the need of LSI in pre-hospital patients. Further inclusion criteria are triage conducted on humans more than five years of age and articles written in English. We ought to investigate if there is a correlation between decreasing HRV and/or HRC and the increased risk of having the need of an LSI.

The following combinations of search terms were used: ("heart rate variability"OR"heart rate complexity") AND"prehospital"OR ("heart rate variability"OR"heart period variability"OR"heart rate complexity") AND (lifesaving interventions OR life-saving interventions). The reference lists for any review articles found in the initial search, as well as citations from eligible studies, were mined for additional studies.

## Results

The comprehensive literature search, conducted with the specified search criteria, resulted in the identification of 18 papers from the MEDLINE database, supplemented by further exploration of references. Each of these papers was meticulously evaluated against our inclusion criteria to ensure relevance and scientific rigor. Of the initially 18 identified studies, two were excluded as they did not constitute original research since they were review articles. Ten studies were excluded as they did not meet our inclusion criteria. Some of which were performed in animals and others in patients who were not in a prehospital setting among these intrapartum females and other hospitalized patients. Moreover, one article was mined and included because it contributes original research related to the prediction of an unfavorable outcome when utilizing HRV in 55 patients [10].

**Fig. 1:**
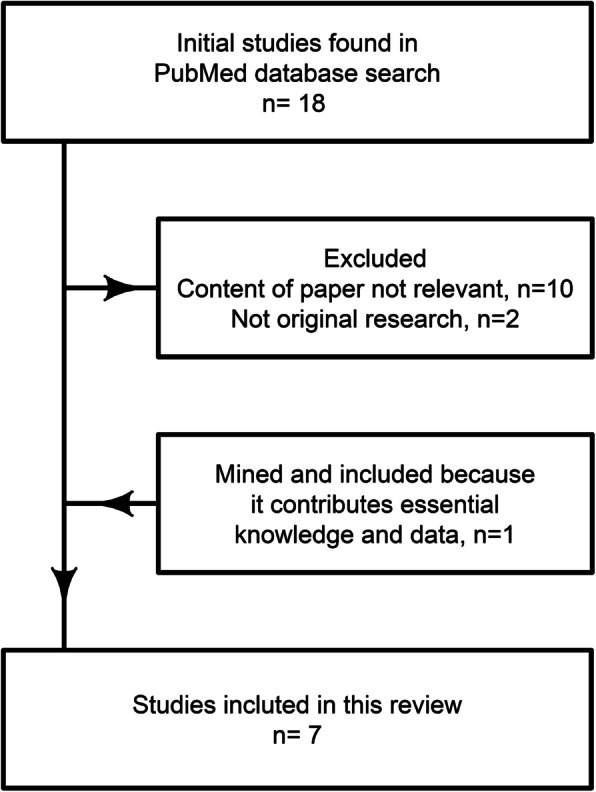
Flowchart of studies included and excluded

Therefore, seven studies met all the inclusion criteria and were deemed suitable for in depth analyses (Fig. 1).

Table 1 comprises information on demography parameters and vital signs used in triage and number of LSI received, when disclosed. There was a larger number of male patients compared to female in all but one study, where the distribution between the sexes was equal. In this study, mean age of those with an unfavorable outcome was higher than those with a favorable outcome. In all other studies, a higher likelihood of receiving an LSI was recognized in younger patients. (Table 1) Regarding race, height, and weight there was no association found between patients who subsequently received an LSI and who did not.

**Table 1:** Characteristics of patients’ demographics, vital signs including HRV/HRC and study endpoint

Study (year)	Numbers of patients in study	Number of males (%) LSI/Non- LSI	Mean age (Years) LSI/Non- LSI	HR (BPM) LSI/Non-LSI	GCS LSI/Non-LSI	Trauma triage score	SBP (mmHg) LSI/Non-LSI	HRV and HRC investigated	Study endpoint and prediction
Cancio et al (2008) [13]	374	77.8/68.8	33.9/38	108.32/99.17	8.10/13.97	ISS	119.48/124.60	HRC & HRV	LSI’s
Liu et al (2014) [8]	104	79	40	93 ± 19	12 ± 5	N/A	135 ± 22	HRV & HRC	LSI’s
Liu et al (2015) [15]	108	76	37	106 ± 27	9 ± 5	ISS	116 ± 26	HRV & HRC	LSI’s
Kumar et al (2019) [4]	225	85.7/65.3	39/44	93.49/85.42	15 (3–15)/15 (15–15) *	T-RTS, MEWS & M-GAP	126.14/138.45	HRV & HRC	LSI’s
Rickards et al. (2010) [12]	159	72/61	34/39	N/A	14.5 ± 0.2/14.7 ± 0.1	ISS	131 ± 5/131 ± 2	HRC	LSI’s
King et al (2009) [5]	75	62.7	47	92	53% with 15 23% with 14 21% with 13	ISS	138	HRV	LSI’s
Yperzeele et al. (2016) [10]	55	38/54.2	72.4/64.9	95 (18)/95 (26)	15 (12–15)/15 (14–15)^*^	N/A	159/157	HRV	Unfavorable outcome

Life-saving interventions (LSI), Glasgow Coma Scale (GCS), Heart Rate (HR), Beats Per Minute (BPM), Systolic Blood Pressure (SBP), Heart Rate Variability (HRV), Heart Rate Complexity

(HRC), T-RTS (Trauma Revised Trauma Score), MEWS (Modified Early Warning Score), and M-GAP (Modified Glasgow Coma Scale Age Pressure)

^*^Median in parentes

In the studies comparing patients who received LSI with those who did not, it was observed that the patients who did not receive an LSI had noticeably lower heart rates and higher systolic blood pressure. Furthermore, in most studies, the Glasgow Coma Scale (GCS) was found to be decreased (< 15), with the lowest scores observed in patients who received LSI.

An LSI event is a composite of different interventions applied to the trauma patient. There is not a universal definition of LSI; hence, this composite event is heterogeneous and varies between the included studies. (Table 2).

**Table 2:** Lifesaving Interventions (LSI’s) included in the studies and other endpoints

Study (year)	Cardiopulmonary Resuscitation	Needle Decompression	Endo-tracheal Intubation	Cardioversion	Blood Transfusion	Cricothyroidotomy	Angioembolization	Thoracotomy	Tube Thoracostomy
Cancio et al (2008) [13]	√	√	√	√		√			
Liu et al (2014) [8]	√	√	√	√	√	√	√	√	√
Liu et al (2015) [15]	√	√	√	√	√	√	√	√	√
Kumar et al. (2019)^*^ [4]	√	√	√	√	√	√	√	√	√
Rickards et al. (2010) [12]	√	√	√	√	√	√	√	√	√
King et al (2009) [5]	“Severely injured”, defined by at least 2 of 3 blinded practicing trauma surgeons								
Yperzeele et al. (2016) [10]	“Unfavorable outcome”, defined as requiring admission to the intensive care unit, mortality during hospitalization, or extended hospital stay (> 30 days) following the emergency intervention								

Torniquets and Pericardiocentesis is not included

^*^ Kumar et al. also included vasoactive medications and hyperosmolar fluid therapy as an LSI. This is not depicted in Table 2

Overall, the results from the studies included, showed that decreased HRV and/or HRC was associated with one or more LSI’s, indifferent of analysis technique. The AUC increased in all the studies when adding HRV and/or HRC to existing trauma triage scores or vital signs such as heart rate (HR), ranging with a ΔAUC between 0.14 to 0.40. (Table 3) In one of the studies statistically significant difference between LSI’s and non-LSI when using HRV was not found, but this was significant when applying a complex analysis technique such as SampEn or ApEn [8].

**Table 3:** Comparison of AUC—values from Receiver Operating Curves (ROC)

Study (year)	**AUC for HR**	**AUC for GCS**	**AUC for HRC/HRV**	**AUC for Combined score**	**∆AUC Lowest to highest AUC**	**Excluded due to noise**	**ECG overview**
Cancio et al. (2008) [13]	N/A	0.80	(SampEn & DFA) 0.76	HRC + GCS: 0.897	0.14	48%	375 Hz EC: < 800 R-R beats, ectopic beats or extensive noise
Liu et al. (2014) [8]	0.73	GCS + HR: 0.92	(SampEn) HRC + GCS: 0.94 (SampEn) HRC + HR: 0.81	HRC + GCS + HR: 0.99 (incl. machine learning)	0.26	12%	230 Hz EC: N/A
Liu et al. (2015) [15]	0.57	0.91	N/A	HRC + HR: 0.86 HRC + GCS: 0.97	0.40	N/A	375 Hz EC: < 15–20 min., no data excluded due to noise
Kumar et al. (2019) [4]	0.61	0.39	(SampEn) 0.75	GCS + HR: 0.65	0.36	0.6%	EC: < 5 min., > 30% ectopy and/or artifacts
Rickards et al. (2010) [12]	N/A	0.800	(FD-L) 0.70	HRV + GCS: 0.897	0.20	None	375 Hz EC: < 800 R-R beats, < 0,5% ectopic beats
King et al. (2009)	0.47—0.68^*^	0.47—0.68^*^	(SDNN) 0.74	N/A	0.25	1%	EC: < 200 QRS, extreme noise
Yperzeele et al (2016) [10]	Only p—values available in the article					None	EC: < 5 min., > 25% of ectopic beats and/or noise

Area Under Curve (AUC), Heart Rate (HR), Glasgow Coma Scale (GCS), Heart Rate Variability (HRV), Heart Rate Complexity (HRC), Electrocardiogram (ECG)

Standard Deviation of Normal-to-Normal R-R interval (SDNN), Sample Entropy (SampEn), Fractal Dimensions by curve Length (FD-L) and Detrended Fluctuation Analysis (DFA)

Excluding criteria (EC)

^*^HR, SBP and GCS measured alone gives an AUC between; 0.47—0.68

One study, found contrary to the other studies that an increase in HRV measured by DFA was associated to LSI’s [4]. In two of the studies the explanation for the increased ΔAUC was due to an increase in negative predictive value, whereas positive predictive value did not increase significantly.

The share of ECG data excluded due to noise was between 34 to 74% in the earlier studies between 2008 and 2010 [12, 13], whereas a study from 2016 found an exclusion rate of only 0.6% of the patients due to ECG noise [10] (Table 3). This corresponds to another study from 2015 where HRV and HRC still were able to discriminate between patients who subsequently received a LSI and those who did not, despite presence of noise on the ECG [15].

## Discussion

The major findings of this review were: 1) Adding HRV and HRC to existing trauma triage scores or vital signs such as heart rate in pre-hospital patients could increase ability to distinguish between patients subsequently received an LSI. 2) Adding HRV and HRC to traditional triage scores could potentially be incorporated using existing hardware. 3) Over time there has become less ECG noise which increases quality of ECG data from four studies.

The patients in the included articles were respectively transported to hospitals in US, Singapore, Brussels. To insure a reliable and representative cohort of included patients, the cohorts were compared to a large study conducted in UK and Germany. This study included 68.510 patients, whereas the mean age was 56.3 ± 22.7, 65.7% of the patients were male [16]. These numbers match with the demography of the patients in the included articles.

Traditional vital signs were not all always useful predictors for a subsequent LSI even though HR to a certain extent were related to LSI [4, 15]. One of the most utilized vital signs included in triage scores is blood pressure, but in the studies included in this review, blood pressure measurement was not found as a predictor for LSI’s. An explanation for this could be the autonomic sympathetic drive compensating found in otherwise healthy persons when experiencing e.g. hemorrhage. The increased sympathetic drive compensating for blood loss could hypothetically be measured as a decrease in HRV and/or HRC. This decrease in HRV and/or HRC could potentially help to identify patients with normal vital signs otherwise getting overlooked as stable, but who subsequently will receive an LSI [12].

Even though no correlations were found between patients who received an LSI and those who did not with normal vital signs, the ∆AUC was 0.10 when adding HRV to the existing trauma triage score comprising vital signs and GCS. This agrees with the included studies, finding heart rate metrics from ECGs beneficial for discriminating between patients who subsequently will receive an LSI and who will not.

The integration of heart rate variability metrics into pre-hospital trauma triage remains unestablished, and a potentially attributing factor for this could be challenges posed by ECG analysis noise. This noninvasive vital parameter is derived from cutaneous electrical leads which cause increased ectopy and artifacts on the ECG, especially in a remote setting or en-route transport. This instigates ineligible ECG waveforms [4], has been noticeable in previous studies, where between 34 to 74% of ECG records were excluded due to noise [12, 13]. Good ECG recording with limited noise is therefore essential before implementing HRV/HRC as a triage parameter. The studies with high share of noise are respectively from 2008 and 2010, whereas a newer article from 2016 established that registration of in-hospital HRV is both feasible and reliable, with an exclusion rate of only 0.6% of the patients due to ECG noise [10] (Table 3). This could indicate that noise reduction and better ECG recording is already developed, which decreases noise on ECG and its influence on analysis.

Other limitations include interindividual variability, chronic diseases and comorbidities e.g. ischemic heart disease and diabetes which has shown to affect HRV and HRC a great deal, but this data was not available in the included studies, hence these must be regarded as unmeasured confounders [5, 12]. The number of patients examined in the included studies ranges from 55 to 374 (Table 1). Larger studies could potentially increase robustness and include a greater variety of comorbidities in the study cohort, hence a more diverse population of patients. This could potentially identify sub-groups were HRV and HRC is more or less useful in triage.

In all the studies included, an analysis of ECG waveforms showed great/high predictive value for triage of pre-hospital patients. The results show an even greater/higher ∆AUC when incorporating this heart rate analysis in ROC. This is supported by the notion that one of the studies found no significant correlation while using HRV but did with HRC. This suggests that this more complex heart rate analysis techniques could achieve even higher predictive value than HRV. This would not pose a limitation, as the ECG metrics remain consistent regardless of the techniques employed.

In the studies included HRV and/or HRC was added to existing triage scores which gives credence to the notion of incorporating the two heart rate variability metrics to the traditional vital signs and not as independent triage scores to distinguish patients who will be needing LSI from patients who will not [5, 12].

A variation of analysis techniques was applied to the ECG metrics to derive HRV and HRC in the studies. The technique showing the greatest independent AUC was SampEn (0,76). SampEn and ApEn are similar analysis techniques computationally, but they still vary a bit and both of them have been included in multiple of the papers. SampEn displays more suitable characteristics for pre-hospital triage hence a decrease in the amount of QRS complexes has relatively little effect on it. SampEn can function with a data set down to approximately 100 beats [13]. While other techniques usually need up to 800 consecutive beats [12].

As aforementioned two studies found high negative predictive values and low positive predictive values. This means that potentially healthy patients were over-triaged. This can turn out to be more costly due to the larger number of patients needing rapid evacuation and the need for LSI according to HRV/HRC. But on the other hand, with a large negative predictive value less patients will be triaged as non-LSI when they need medical interventions.

## Conclusion

In this review both HRV and HRC increased AUC in prediction of an LSI when added to existing trauma triage scores or vital signs and HRC showed better potential than HRV. HRV and HRC could both be derived from existing ECG data obtained from pre-hospital patients and potentially with pre-existing hardware.

ECG data is indispensable in order to calculate HRV and HRC and over time the share of available ECG data for analyses has increased.

## Data Availability

No datasets were generated or analysed during the current study.
